# The impact of COVID-19 on clinical research: the PIPPRA and MEDRA experience

**DOI:** 10.12688/hrbopenres.13283.1

**Published:** 2021-05-18

**Authors:** Louise Larkin, Tala Raad, Anusha Moses, Alexander Fraser, Stephen Gallagher, Bente Appel Esbensen, Liam Glynn, Anne Griffin, Audrey C Tierney, Norelee Kennedy

**Affiliations:** 1 School of Allied Health, Faculty of Education & Health Sciences, University of Limerick, V94 T9PX, Ireland; 2 Health Research Institute, University of Limerick, Limerick, V94 T9PX, Ireland; 3 Health Implementation Science and Technology, Health Research Institute, University of Limerick, V94 T9PX, Ireland; 4 School of Medicine, Faculty of Education & Health Sciences, University of Limerick, V94 T9PX, Ireland; 5 Department of Rheumatology, University Hospitals Limerick, Limerick, Ireland; 6 Department of Psychology, Faculty of Education & Health Sciences, University of Limerick, Ireland; 7 Copenhagen Center for Arthritis Research, Center for Rheumatology and Spine Diseases, Rigshospitalet, Glostrup, Denmark; 8 Department of Clinical Medicine, Faculty of Health and Medical Sciences, University of Copenhagen, Copenhagen, Denmark; 9 HRB Primary Care Clinical Trials Network Ireland, Limerick, Ireland

**Keywords:** Clinical research, rheumatology, COVID-19, pandemic, feasibility

## Abstract

**Background**: Coronavirus disease 2019 (COVID-19) has had a significant impact on clinical research. This paper aims to provide an insight into how the COVID-19 pandemic, associated public health restrictions and international guidance on the conduct of clinical research impacted two clinical rheumatology research trials - the Physiotherapist-led Intervention to Promote Physical Activity in Rheumatoid Arthritis (PIPPRA) and the MEDiterranean diet in Rheumatoid Arthritis (MEDRA) projects.

**Methods**: The March 2019 public health restrictions imposed to mitigate the risk of COVID-19 occurred at a time when PIPPRA was in the process of delivering assessment and intervention on a face-to-face basis (n=48) and MEDRA had commenced recruitment. Participants in PIPPRA and MEDRA had a diagnosis of rheumatoid arthritis, with some being immunosuppressed and thus at a higher risk for COVID-19. The decision-making processes of both trials is outlined to demonstrate the required amendments to continue in the context of the COVID-19 pandemic.

**Results**: Amendments to PIPPRA and MEDRA trial protocols were agreed and received ethical and funder approval. Both trials switched from a face-to-face delivery to a telehealth using online platforms. The PIPPRA study was paused for five months (April-August 2020), resulting in n=33 (60%) deviations from assessment protocol. MEDRA switched from face-to-face to online recruitment with 20% (n=35/44) deviation in recruitment. Of the n=18 participants who consented to participating in a face-to-face trial, just n=2 (11%) opted to engage with telehealth delivery of the intervention. MEDRA assessment and intervention deviations were 100% as no sessions were completed as planned in 2020.

**Conclusions**: The COVID-19 pandemic has severely impacted the PIPPRA and MEDRA clinical trials. Moving face-to-face clinical research to telehealth delivery may not be the panacea it is purported to be. Our experiences may be of benefit to researchers, clinicians, and funders in seeking to continue clinical research during a global pandemic.

## Introduction

Severe acute respiratory syndrome coronavirus 2 (SARS-CoV-2) causing coronavirus disease 2019 (COVID-19) has had a significant impact on global healthcare, in both delivery of healthcare services and in clinical research ( Marcum *et al.*, 2020; Noor *et al.*, 2020; Stiles-Shields *et al.*, 2020). The COVID-19 pandemic and its associated public health restrictions has impacted clinical research and researchers and participants ( European Commission *et al.*, 2020) wherein extraordinary measures, such as adjustment of trial methodology, has been required to deal with this impact ( European Commission *et al.*, 2020). Ongoing participant recruitment, visits to healthcare facilities, trial staff availability and continued involvement of participants in the trial are some elements that need to be considered in the current clinical research environment ( European Commission *et al.*, 2020). Authors in the fields of oncology, gastroenterology and paediatrics have documented the impact of COVID-19 on their clinical research ( Marcum *et al.*, 2020; Noor *et al.*, 2020; Stiles-Shields *et al.*, 2020). However, the impact of COVID-19 on rheumatology clinical research remains undocumented to our knowledge.

Rheumatoid arthritis (RA) is a chronic autoimmune inflammatory condition that affects 0.5% of the adult population worldwide ( Carmona *et al.*, 2010). RA affects females three times more than males, with a peak incidence after 40 years of age ( Carmona *et al.*, 2010). RA is characterised by swollen and painful joints, and clinical symptoms such as fatigue. RA is diagnosed using the American College of Rheumatology/European League Against Rheumatism (EULAR) 2010 criteria ( Wolfe *et al.*, 2010). In the Republic of Ireland, people who have RA are categorised within a high risk category for COVID-19, due to immunosuppression ( Health Products Regulatory Authority, 2020; Health Service Executive, 2020), although this categorisation contrasts with EULAR’s provisional recommendations ( Landewé *et al.*, 2020). The World Health Organisation declared COVID-19 to be a pandemic on 11 ^th^ March 2020 ( Livingston *et al.*, 2020). Two rheumatology clinical research projects were ongoing through the University of Limerick, Ireland, when the first case of COVID-19 was diagnosed on the island of Ireland on 26 ^th^ February 2020 ( Perumal *et al.*, 2020). This was quickly followed by the confirmation of the first case of COVID-19 in the Republic of Ireland on 29 ^th^ February 2020.

The two clinical research projects were the Physiotherapist-led Intervention to Promote Physical Activity in Rheumatoid Arthritis (PIPPRA) and the MEDiterranean diet in Rheumatoid Arthritis (MEDRA) projects. PIPPRA is a pilot randomised control feasibility study. The project is funded through the Health Research Board Definitive Intervention and Feasibility Awards 2018 (DIFA-2018-004). The MEDRA project is a pilot randomised control (RCT) study. Both projects are collaborations between the University of Limerick and University Hospitals Limerick Rheumatology department, with national and international collaborators from the healthcare, patient, research, and academic fields. Here the authors outline the impact of the COVID-19 pandemic on the PIPPRA and MEDRA projects, providing an insight into how the pandemic impacted two clinical research projects in rheumatology within an Irish context. This paper contributes to a very limited international body of literature on clinical research during the COVID-19 pandemic and seeks to inform academics, researchers, and clinicians about the considerations for conducting clinical research during a global pandemic. This paper aims to demonstrate how the COVID-19 pandemic, associated public health restrictions and international guidance on the conduct of clinical research impacted two clinical rheumatology research trials.

### Protocol design

The PIPPRA feasibility study was designed following extensive work in intervention development, guided by the Medical Research Council complex intervention framework ( Craig *et al.*, 2008). This development work included systematic reviews, physical activity monitor validation, and qualitative research with people who have RA and healthcare professionals ( Larkin *et al.*, 2016a; Larkin *et al.*, 2015a; Larkin *et al.*, 2015b; Larkin & Kennedy, 2014; Larkin *et al.*, 2016b; Larkin *et al.*, 2017). The aim of this pilot RCT is to examine the feasibility of a physiotherapist-led, behaviour change theory informed, physical activity intervention to promote physical activity in people who have RA who have low levels of physical activity. The primary deliverables of the PIPPRA pilot study are to determine the rate of recruitment to the study and the acceptability of the intervention to participants as well as test the feasibility of the intervention on secondary disease focused outcome measures.

The MEDRA project is a 12-week pilot randomised, controlled dietary intervention study in Irish adults living with RA. The MEDRA project aims to explore the effect of implementing either a Mediterranean type diet (MED Diet) or the Irish Healthy Eating Guidelines on the inflammatory profile, disease activity and quality of life in adults living with RA in Ireland. The MEDRA project will provide data on the habitual dietary intakes of adults with RA in Ireland and their baseline adherence to key components of either the MED Diet or the Healthy Eating Guidelines. MEDRA will also evaluate the effects of implementing the traditional Mediterranean diet adapted for the Irish culture compared to the Irish Healthy Eating Guidelines on inflammatory profile, disease activity and quality of life of adults with RA. With its specific design, the MEDRA study aims to identify the attitudes, beliefs, and perceived barriers towards adhering to a MED Diet in an Irish context and under dietetic supervision.

The clinical research community was not prepared for the disruption caused by the COVID-19 pandemic, which has impacted the operations and conduct of trials globally ( Bierer *et al.*, 2020). The COVID-19 pandemic has presented both challenges and opportunities to rheumatologists and rheumatology health professionals who have rapidly adopted telemedicine into routine practices ( American College of Rheumatology, 2020). Adaptations in clinical practice have been mirrored in clinical research; however, clinical research in particular was severely impacted in the early months of the COVID-19 pandemic ( Tuttle, 2020). Changes in clinical research conduct have been rapidly introduced to prioritise participant and researcher safety and to accommodate the requirement for social distancing to slow the spread of the virus ( Bierer *et al.*, 2020). Such changes, e.g. trial suspensions, were made after rapid consideration of whether risks could be mitigated and whether trial and data integrity could be assured ( Bierer *et al.*, 2020). This paper seeks to outline the experience of two clinical rheumatology research projects in the Republic of Ireland across the first 10 months (March 2020 – December 2020) of the COVID-19 global pandemic.

## Methods of trials

The PIPPRA study protocol is detailed on the Clinicaltrials.gov registry (Identifier: NCT03644160, August 23 2018). Ethical approval was obtained from the Health Service Executive Research ethics committee University Hospitals Limerick for the original study protocol in May 2019. The MEDRA Study protocol is detailed on the Clinicaltrials.gov registry (Identifier: NCT04262505, February 10 2020). The protocol has been approved by the Education and Health Sciences Research Ethics Committee at the University of Limerick (2020_09_05_EHS) and by the Health Service Executive Mid-Western Regional Hospital Research Ethics Committee (REC Ref 103/19).

### Inclusion criteria

Inclusion criteria for PIPPRA are as follows; participants aged 18 years or older, rheumatologist confirmed diagnosis of RA, independently mobile and low levels of physical activity as categorised on the Godin-Shephard Leisure-time Physical Activity Questionnaire ( Godin, 2011). PIPPRA exclusion criteria are use of a mobility aid, not fluent in English, already physically active as per the Godin-Shephard Leisure-time Physical Activity Questionnaire ( Godin, 2011). Eligible participants for the MEDRA study included those who are aged ≥ 18 years with a definite diagnosis of RA according to American College of Rheumatology (ACR)/European League Against Rheumatism (EULAR) criteria ( Aletaha *et al.*, 2010). Participants are excluded if they have commenced any nutritional supplements or a new dietary regime in the month prior to study enrolment. Women who are pregnant, breastfeeding or planning to become pregnant are also not included.

Participants for both the PIPPRA and MEDRA projects were originally recruited from two hospital rheumatology clinics, one urban and one rural. Both assessment and intervention sessions for PIPPRA and MEDRA were to be delivered in person at a clinical research facility, located in the grounds of a large urban hospital, as per the study protocol.

### Recruitment and assessment

The target recruitment rate for PIPPRA was four participants per month for one year (N=48). A target of 40 participants, with 20 participants in both the control and intervention groups, for completion of the study was set. This sample size is expected to provide sufficient data to meet the primary outcomes of the PIPPRA project. A definitive sample size of a large-scale RCT will be determined from the results of this trial. PIPPRA recruitment commenced on 1 ^st^ October 2019. Assessments are conducted at baseline, eight and 24 weeks (total assessments N=144). Assessments are conducted by independent assessors (LL – physiotherapist; MR - research manager) who are blinded to participant allocation. Assessments consist of a battery of self-report questionnaires (self-report physical activity - Yale Physical Activity Survey; behaviour change - Theory of planned behaviour questionnaire; quality of life - Quality of Life in Rheumatoid Arthritis; pain – visual analogue scale over past seven days; fatigue - Bristol RA Fatigue Multi-dimensional Questionnaire; functional ability - Health Assessment Questionnaire; sleep – Pittsburgh Sleep Quality Index), objective physical activity measurement using the ActivPAL™ activity monitor and disease activity (Disease Activity Score-28). DAS-28 requires blood sample collection for erythrocyte sedimentation rate (ESR) to calculate the DAS-28 score. Blood sample collection is conducted by a research nurse. Assessments for PIPPRA were to be completed by March 2021.

Recruitment for MEDRA commenced on 1 ^st^ February 2020. The target sample size is (N=44), allowing for a 10% drop-out rate over the 12-week study period. Participants in the MEDRA study are expected to attend three face-to-face appointments at baseline (week 0), mid-intervention (week 6) and post-intervention (week 12). All study appointments are to be conducted with the study Registered Dietitian (RD) at the same research facility as the PIPPRA study. Study appointments for both diet groups include a nutritional educational session on how to adopt the key principles of either target diets, anthropometric measurements including height, weight, waist circumference and mid-upper arm circumference as well as blood sampling for ESR, C-reactive protein (CRP), tumour necrosis factor alpha (TNF-α), interleukin 6 and 10, performed by a qualified research nurse. Given that the recruitment of participants and study appointments were to run simultaneously; it was expected that data collection for the MEDRA study would be completed by October 2020.

### Intervention

PIPPRA participants are randomised to the intervention or control group. Randomisation is conducted independently, by a biostatistician (AS) and project research assistant (AM, TP). Participant allocation is concealed to both assessors and sessional physiotherapists. Intervention sessions are delivered by two physiotherapists (LH, LK). Sessional physiotherapists are blinded to assessment data. The intervention group receive four 1:1 60-minute sessions (total target intervention sessions N=80). The intervention is delivered over eight weeks, with one session in Week 1, Week 3, Week 5, and Week 7. Intervention and control groups receive a physical activity information leaflet, which outlines the physical activity guidelines for people who have RA ( Rausch Osthoff *et al.*, 2018).

MEDRA participants are randomly allocated to either a MED Diet group or the Healthy Eating group using a permuted block randomisation scheme with 1:1 ratio allocation. Randomisation is conducted by an independent statistician at the University of Limerick, who is not involved in the conduct of the RCT. To guarantee concealment of allocation, participants are randomised once written informed consent has been obtained. As with most dietary intervention studies and for the purpose of the MEDRA study, blinding to intervention is not possible. All study appointments were to be conducted with the study Registered Dietitian (RD) at the same research facility as the PIPPRA study. Study appointments for both diet groups includes a nutritional educational session on how to adopt the key principles of either diet intervention. Participants in both intervention groups are provided with printed resources to inform participants of the principles of the assigned diet as well as food hampers to demonstrate each eating pattern.

## Amendments to protocol due to COVID-19 pandemic

**Figure 1.**
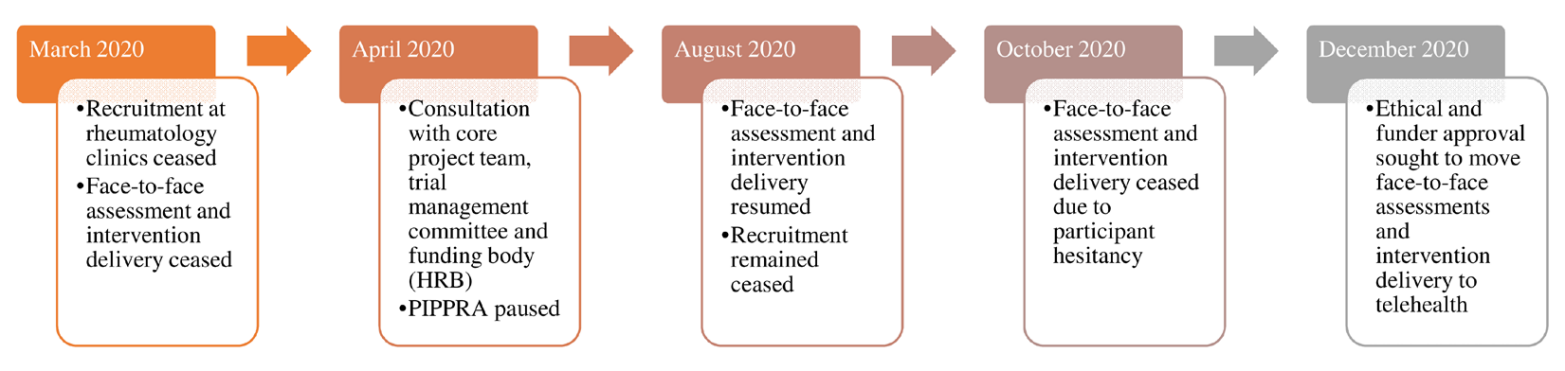
Overview of PIPPRA project during COVID-19 pandemic 2020. PIPPRA, Physiotherapist-led Intervention to Promote Physical Activity in Rheumatoid Arthritis; COVID-19, coronavirus disease 2019; HRB, Health Research Board.

**Figure 2.**
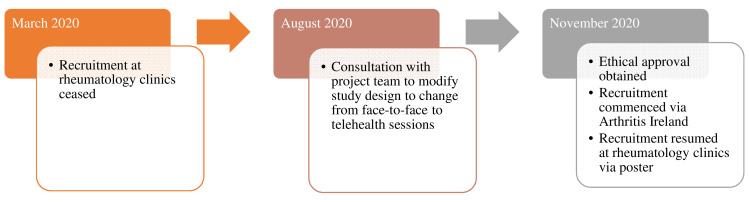
Overview of MEDRA project during COVID-19 pandemic 2020. MEDRA, MEDiterranean diet in Rheumatoid Arthritis; COVID-19, coronavirus disease 2019.

Due to the COVID-19 pandemic, public health restrictions and participant hesitancy to attend in-person appointments, changes to both the PIPPRA and MEDRA protocols were necessary. Here the authors outline the amendments to study protocol that were implemented in each RCT ( Figure 1 and Figure 2). Amendments were considered and implemented in light of participant and research staff safety, public health restrictions and published guidance on the conduct of clinical research trials during the COVID-19 pandemic ( European Commission *et al.*, 2020; Health Products Regulatory Authority, 2020). The decision-making process for PIPPRA and MEDRA varied.

In the case of the PIPPRA study, the Primary Investigator (NK) consulted with the core project team and the trial management committee regarding the concerns of continuing to conduct face-to-face clinical research during the pandemic. The trial management committee consists of the primary investigator (NK), four project collaborators (AF, LG, SG, LL) one research nurse (EC), one biostatistician (AS) and one research assistant (AM). All trial management committee members are based in the mid-West of Ireland. Consultation also occurred with the funding body, the Health Research Board (HRB), on the conduct and potential pausing of the PIPPRA project.

The MEDRA project is co-led by a PhD student (TR) and PhD Primary Supervisor (AT). Thus, the decision-making process in MEDRA’s case was at a local level but had the same considerations as the PIPPRA study. The Health Products Regulatory Authority (HPRA) guidance (2020) that the safety of subjects (participants) is of primary importance was at the core of the decision-making process for both the PIPPRA and MEDRA studies. In particular, the HPRA specifically identified subjects who are immunosuppressed, including some people who have RA, as an at-risk group and stated that careful consideration should be given to continuing with clinical trials involving such subjects.

### PIPPRA amendments

The PIPPRA project was paused for four months from April 2020 to August 2020. The PIPPRA project resumed on a face-to-face basis in August 2020. PIPPRA participants attended for assessment and intervention face-to-face until late October 2020. From October 2020 onwards participants were hesitant to attend face-to-face sessions, coinciding with the Republic of Ireland’s second lockdown which commenced on 22 ^nd^ October 2020. In December 2020, the PIPPRA project sought ethical and funder approval to move assessments and intervention delivery to virtual platforms, or telephone audio where participants were unable/unwilling to access a virtual platform. The proposed virtual platform was Microsoft Teams. Subsequently participants requested to use other virtual platforms, namely Zoom and Whatsapp, to access video calls. These requests were facilitated. Access to virtual platforms proved challenging for some participants who were not familiar with videocalls. To enhance engagement, the PIPPRA research assistants (AM, TP) conduct a trial video call with intervention participants, prior to session 1 of their intervention. Participants expressed a preference for phone audio calls for assessments and this request was then facilitated. Changing assessments to a telehealth/audio platform also required revision in the assessment methods. The DAS-28 was removed from the assessment, due to an inability to collect a blood sample. The ActivPAL activity monitor was posted to participants, instead of being applied by the assessor (LL) in a face-to-face assessment. The postal package included an information leaflet on ActivPAL application and wear instructions and Tegaderm for application. Participants were advised on ActivPAL application by the assessor (LL) during the assessment. ActivPALs are set to record from the date and time of the assessment. Participants returned the ActivPAL by post to the assessor (LL). Thus, there was minimal additional burden on ActivPAL application and return.

### MEDRA amendments

Recruitment and assessment of participants became very difficult given the ongoing COVID 19 public health situation. In addition, many concerns that were raised by recruited participants regarding their discomfort at the idea of coming to the hospital for appointments. Therefore, the MEDRA research team decided to modify the study design in August 2020 and to deliver the dietary intervention via telehealth methods through Zoom meetings. The rationale for shifting to a web-based/telehealth approach is that it is a safe, convenient, and potentially the most inclusive way to collect data and capture diverse dietary perspectives across Ireland while avoiding face-to-face contact with participants. Shifting to telehealth, however, also meant rethinking those primary outcome measures of the study that involved blood sampling and anthropometric assessment. In November 2020 and upon obtaining ethical approval for the new study design, recruitment of study participants resumed through a collaboration with the patient organisation ‘Arthritis Ireland’ and through their social media platforms. This new collaboration facilitated access to prospective participants. Recruitment through outpatient rheumatology clinics in University Hospital Limerick resumed through poster advertisement only.

## Results

Our results outline the impact that the COVID-19 pandemic has had on participant recruitment, assessment, and intervention delivery.

### Recruitment

PIPPRA recruitment commenced at rheumatology clinics on 1 ^st^ October 2019. MEDRA recruitment commenced at rheumatology clinics on 1 ^st^ February 2020. Recruitment at rheumatology clinics ceased on 3 ^rd^ March 2020. At the time of recruitment cessation, n=48 participants had consented to participation in the PIPPRA study. PIPPRA had reached its recruitment target in six months. MEDRA had recruited 18 participants at the time of recruitment cessation. MEDRA switched from face-to-face to online recruitment with 20% (n=35/44) deviation in recruitment.

### Assessments

**Figure 3.**
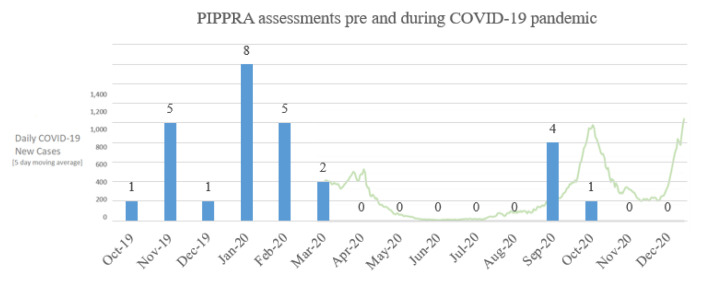
PIPPRA assessments completed pre and during the COVID-19 pandemic. Source for daily COVID-19 case data: #COVIDWATCHIRL via https://covidwatcheu-npa.shinyapps.io/covid/?covidtrends. PIPPRA, Physiotherapist-led Intervention to Promote Physical Activity in Rheumatoid Arthritis; COVID-19, coronavirus disease 2019.

As of December 2020, n=20 participants have commenced in the PIPPRA study, n=16 are awaiting baseline assessment (attempting to schedule), n=6 withdrew and n=6 were lost to follow-up prior to baseline. Pre-trial amendments, the PIPPRA trial protocol planned for the delivery of n=55 assessments for participants who had commenced in the study. N=22 assessments were conducted between October 2019 and March 2020 ( Figure 3). N=5 assessments were conducted between August and October 2020 ( Figure 3). No assessments occurred in November-December 2020 due to participant hesitancy in attending for assessment with increased public health restrictions. The impact of COVID-19 restrictions resulted in n=33 (60%) deviations from assessment protocol.

**Figure 4.**
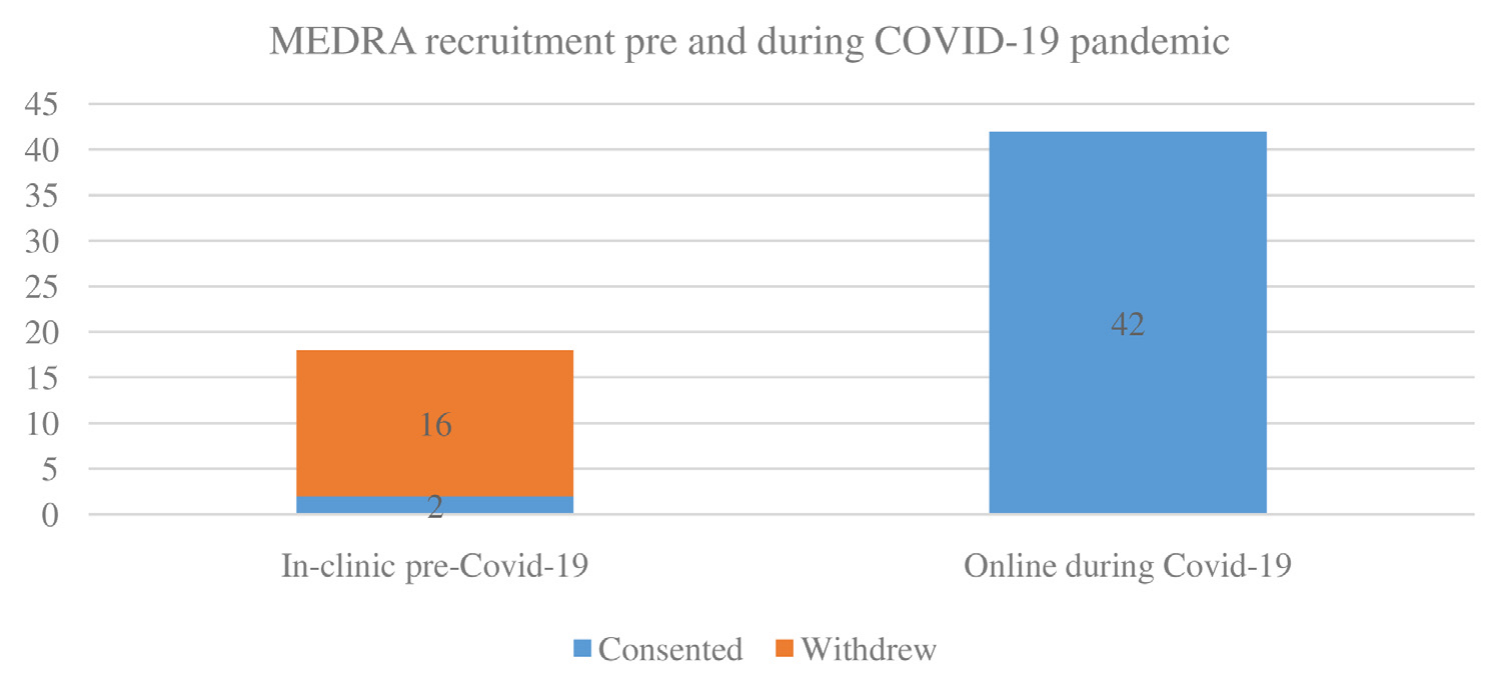
MEDRA recruitment. MEDRA, MEDiterranean diet in Rheumatoid Arthritis; COVID-19, coronavirus disease 2019.

For MEDRA n=18 participants who had consented to participation at face-to-face clinics were awaiting baseline assessments in March 2020. Of these n=18 participants, only n=2 agreed to proceed with telehealth delivery of the dietary intervention ( Figure 4). N=16 were lost, primarily due to limited technological literacy. The two participants who agreed to participate in the telehealth delivery of the study had full access to a smartphone and the internet. N=42 were recruited through Arthritis Ireland, the national arthritis patient organisation, between November 2020 and January 2021. All recruited participants had full access to a laptop or a smartphone. No assessment sessions were conducted in 2020, resulting in a 100% deviation from protocol.

### Interventions completed

Trial protocol planned for the delivery of n=36 intervention sessions for participants who had commenced in the PIPPRA study. N=26 intervention sessions were delivered between November 2019 and March 2020. N=6 intervention sessions were conducted between August and October 2020. No intervention delivery occurred in November-December 2020 due to participant hesitancy in attending for intervention with increased public health restrictions. The impact of COVID-19 restrictions resulted in n=10 (27%) deviations from intervention delivery protocol. In the MEDRA project, no intervention sessions had been completed by March 2020. No intervention sessions were conducted in 2020, resulting in a 100% deviation from MEDRA intervention protocol. N=40 intervention sessions were conducted between January and March 2021, a one-year deviation from original protocol.

## Discussion

### Summary of main findings

This paper presents the impact of the COVID-19 pandemic on two rheumatology clinical research projects. Significant challenges arose during the first ten months of the projects that necessitated changes to original trial protocols, which resonates with the experiences of clinical research globally ( Bierer *et al.*, 2020). This impacted trial recruitment, participant assessment and intervention delivery and necessitated changes in trial protocol to ensure that clinical research could progress in a safe and acceptable manner for both research participants and trial staff. Our paper provides an insight into the trial management issues that arose in conducting clinical research during a global pandemic. These issues spanned the entire research project, from recruitment, to assessments and intervention delivery.

### Comparison with existing literature

 Among our patient population of people living with RA, participant hesitancy and/or fear about attending face-to-face clinical research session in a clinical research facility located adjacent to an acute hospital proved to be a significant barrier to proceeding with PIPPRA and MEDRA as per the original trial protocols. COVID-19 fear has been reported to impact mental health through traditional and social media coverage which is unrelenting ( Su *et al.*, 2021). The impact of COVID-19 on mental health has been widely reported, with some research reporting that it has negatively impacted approximately 70% of people living with chronic conditions ( Chudasama *et al.*, 2020). Indeed, the authors of that global survey also found that changes in healthcare services for health issues other than COVID-19 had moderate or severe effects on patients since the COVID-19 outbreak ( Chudasama *et al.*, 2020). Worry about contracting COVID-19 was high amongst people with arthritis, with a higher proportion of self-isolation amongst people who have arthritis due to their condition compared to others their age ( Glintborg *et al.*, 2021). In our experience, some of the MEDRA and PIPPRA participants’ hesitancy and COVID-19 fear around engaging in clinical research was influenced by family members, including adult children.

### Strengths and limitations

This novel paper outlines the process of how two clinical research trials responded to the challenge presented by the COVID-19 pandemic to the conduct of clinical research. The decision-making process and amendments implemented to the PIPPRA and MEDRA trials are clearly outlined. The strategies implemented provide information for future clinical research and highlight the importance of risk assessment and risk management strategies in the planning of clinical research trials. The authors could not have foreseen the impact a global pandemic would have on the PIPPRA and MEDRA studies. However, the experiences of both trials highlight the need to prepare for alternative methods of trial delivery, and to plan for the implications of moving a face-to-face trial to telehealth and vice versa. This paper addresses the first 10 months of the global pandemic and does not report on the effectiveness of amendments made to the PIPPRA and MEDRA trials. The results of the impact of amending both trials will be analysed and reported in the future, at which point the authors will evaluate if the steps taken were appropriate and effective.

### Implications for research and policy

Recruitment strategies for both studies required amendments. It became apparent for the PIPPRA and MEDRA studies that participants who had originally consented to participation due to the pandemic were uncontactable or unwilling to engage with telehealth delivery of the study. This was particularly evident in the MEDRA study where 89% (n=16) withdrew from telehealth delivery of project. Thus, additional recruitment strategies were implemented. Virtual recruitment has significant a benefit; that recruitment is not restricted to one locality or rheumatology service. This could be considered a more inclusive recruitment strategy and result in greater transferability of results from the MEDRA study. In PIPPRA’s case, additional recruitment occurred via clinicians engaged in the rheumatology service clinics who referred interested patients to the PIPPRA research team. In February 2021, face-to-face clinics were limited to high priority cases and operated at just 25% capacity of a typical clinic. Most patients attending such clinics did not meet PIPPRA study inclusion criteria. Telephone clinics were run by the rheumatology clinical nurse specialists who assisted the PIPPRA team in study recruitment. This experience demonstrates the importance of effective and collaborative research-clinical partnerships ( Albert *et al.*, 2019), where clinicians act as important gatekeepers to potential research participants. In MEDRA, although limited to people with access to social media accounts, recruitment through social media platforms led to a larger reach of people living with RA and recruitment was highly successful. Thus, consideration must be given to recruitment locations for future research, given that those who engage in clinical research studies may be dependent on this recruitment site and also the mode (face-to-face or telehealth) by which clinical research is conducted. Furthermore, those who engage with clinical research are likely to be highly interested and motivated to engage, and thus transferability of results must be considered in that regard.

Protocol and method changes were required in both PIPPRA and MEDRA. Such changes required ethical approval, and in the case of PIPPRA, funder approval also. In the Republic of Ireland, local research ethics committees responded promptly to requested changes to previously approved clinical research trials. The need to amend trial protocols was recognised and indeed advocated for internationally ( European Commission *et al.*, 2020). The required changes brought new trial management challenges. As previously stated, participant withdrawal due to no not wanting to or not being able to engage with virtual clinical research was an issue. Some previously enrolled participants in the PIPPRA study requested telephone audio calls only, which proved difficult for participants who were in the intervention group. From an assessment perspective, the collection of objective physical activity data was facilitated through the posting of the ActivPAL3™ activity monitor to participants. This required a reallocation of budget funds, as it was not a project cost that was required in the original trial protocol.

Communicating with participants posed a significant challenge for the PIPPRA project. For example, some participants had consented and enrolled in January/February 2020 but were difficult to contact after the official resumption of the project in August 2020. This included participants not responding to phone calls or text messages or phone numbers no longer being in use. This aligns with the experience of communicating with patients in clinical practice during the pandemic. Bos *et al.* (2021) reported that 57% of rheumatologists in Denmark had difficulty in reaching patients (i.e., phone not being answered). It is plausible that patient priorities have changed during the global pandemic. Participating in a clinical research trial may have been a priority in early 2020; however, competing demands, e.g. working from home, managing home-schooling, pandemic fatigue or simply lack of interest due to the wider situation, may have impacted participant motivation to engage with the PIPPRA and MEDRA studies.

Trial assessments required changes due to the switch from face-to-face assessment to phone/telehealth assessment. The DAS28 was removed from both the PIPPRA and MEDRA assessments, as examination of swollen or tender joints and collection of blood samples to calculate the score was not possible. The consequence of this is that an objective measure of disease activity will be absent from the analysis of effectiveness of each intervention on disease activity and should be examined in future research if possible. The challenges of conducting such assessments have been acknowledged from the clinical practice perspective by the American College of Rheumatology, who highlighted the practical difficulties for rheumatology clinicians due to the lack of a direct musculoskeletal exam as well as inability to remotely monitor changes in the musculoskeletal exam over time ( American College of Rheumatology, 2020). Challenges that accompany the use of telehealth/telehealth sessions included the assessment of RA disease activity and inflammatory status, both of which are fundamental to the management of RA and are frequently included as outcome measures in rheumatology clinical research studies. However, the inclusion of patient-reported outcomes (PROs) can greatly enhance a telehealth assessment. The use of PROs has this far been a positive experience in the PIPPRA and MEDRA projects and is easily conducted via telephone and telehealth assessments. PROs are a form of clinical outcome assessment method that complement biomarkers, measures of morbidity (e.g. stroke, myocardial infarction), burden (e.g. hospitalisation), and survival that are used and reported in clinical trials and non-randomised studies ( FDA, 2018). The use of PROs in clinical research has the potential to benefit patients and the wider society ( Rivera *et al.*, 2019). The use of PROs is a positive element of virtual healthcare delivery that was highlighted by a recent review, which reported structured questionnaires and wearable activity trackers could have a greater role in patient care in the future ( Solomon & Rudin, 2020). In the case of PIPPRA, the use of PROs and the ability to provide wearable physical activity trackers has proved easy to facilitate both face-to-face and through telehealth/phone assessments. Observations from the MEDRA participants include that the telehealth method is flexible, more convenient and a resource-saving solution for dietary clinical research. It should be noted that current MEDRA participants are largely comfortable with the use of online platforms. It should be considered that a particular subset of patients who have RA may not be familiar with online platforms and may find completing study questionnaires online a burden and very time consuming. For the PIPPRA study, intervention delivery via telehealth sessions is yet to be evaluated but for the MEDRA study this mode of delivery has been operating effectively. This aligns with the Australian position paper which states that videoconference‐delivered nutrition care is as effective as similar programs conducted in-person ( Kelly *et al.*, 2020). A final consideration is that although the PIPPRA and MEDRA trial staff did not complete additional training to conduct assessment and deliver intervention via telehealth, there may be a need for the provision of training or upskilling opportunities for research staff and clinicians to effectively deliver telehealth.

## Conclusion

This paper aims to provide an insight into how the COVID-19 pandemic, associated public health restrictions and international guidance on the conduct of clinical research impacted two clinical rheumatology research trials. The COVID-19 pandemic has had a significant impact on the delivery of the PIPPRA and MEDRA projects. Feasibility study outcomes, including participant recruitment and retention rates, and study delivery as per protocol, have been severely affected due to the COVID-19 pandemic. Participant reluctance to attend face-to-face sessions demonstrates the need to consider alternative methods of delivery, e.g. telehealth delivery of interventions, where attending in person is not acceptable to participants, in future studies. The optimal balance between telehealth and face-to-face appointments remains uncertain in clinical research for an RA cohort and one option would be to explore a hybrid model of delivery in future research. The need for risk management strategies in clinical research is more evident than ever before. Researchers, clinical partners and funding bodies should ensure that appropriate strategies are identified in advance of project commencement to deal with the potential challenges to original trial protocols. Such risk management should serve to future proof clinical research in an evolving global health situation.

## Data availability

All data underlying the results are available as part of the article and no additional source data are required.
