# Supplementary material for: The impact of COVID-19 on clinical research: the PIPPRA and MEDRA experience
Source: HRB Open Res. 2023 Nov 10;4:55. Originally published 2021 May 18. [Version 2] doi: 10.12688/hrbopenres.13283.2 (PMC10767248; doi:10.12688/hrbopenres.13283.2)
Supplement: Peer review history

# Reviewer report: Reviewer response for version 2

**DOI:** 10.21956/hrbopenres.15149.r36990

**Published:** 2024-01-04

**Authors:** James P. Gavin

**Affiliations:** 1 School of Health Sciences, University of Southampton, Southampton, UK

I congratulate the authors in responding to my review comments. I have no further issues, aside from that the manuscript needs proof-reading as there are a few remaining typographical errors.

Is the work clearly and accurately presented and does it cite the current literature?

Partly

If applicable, is the statistical analysis and its interpretation appropriate?

I cannot comment. A qualified statistician is required.

Are all the source data underlying the results available to ensure full reproducibility?

No

Is the study design appropriate and is the work technically sound?

Partly

Are the conclusions drawn adequately supported by the results?

Yes

Are sufficient details of methods and analysis provided to allow replication by others?

Partly

Reviewer Expertise:

Musculoskeletal health; physical activity; biomechanics; allied health profession education; occupational therapy; rheumatology

I confirm that I have read this submission and believe that I have an appropriate level of expertise to confirm that it is of an acceptable scientific standard.

# Reviewer report: Reviewer response for version 1

**DOI:** 10.21956/hrbopenres.14458.r33662

**Published:** 2023-05-17

**Authors:** James P. Gavin

**Affiliations:** 1 School of Health Sciences, University of Southampton, Southampton, UK

The submitted manuscript reports on the experiences of clinical-academic researchers in the delivering of two rheumatology trials in Ireland during the COVID-19 pandemic. Although the manuscript is novel and interesting to the readership of HRB Open Research, the research methodology is unorthodox, and consequently confusing for the reader at times. For example, descriptive information on the respective trials (MEDRA and PIPPRA) is repeated throughout often without contributing to the specific narrative. Also, I do think the authors could strengthen their submission by reconsidering their use of figures, instead of very basic figures (e.g. Figure 4), which can be succinctly stated in the text, they should strongly consider using PRISMA flowcharts for the respective trials, specifically indicating participant flow through: recruitment, assessments, and interventions completed, respectively.

 Overall, the paper is written in plain English and offers important learning points and implications for clinicians and academics. My specific comments can be found below:

**Abstract**

 The use of the word ‘deviations’ is overly vague (especially in the manuscript Results), instead the authors should succinctly state whether the deviation was negative (a decrease) or positive (an increase in this case).

 The Conclusion is weak and reveals little of what was actually found for the individual trials.

**Introduction**

 Paragraph 1 – there is an over-reliance on the reference ‘European Commission et al’, which arises three times in the opening paragraph. Also, check that this reference style is correct; should it be ‘et al’? I suspect not.

 Paragraph 4 (Protocol design), lines 7-9 – six references are listed to highlight the development work for the PIPPRA trial. Individual references should instead be attributed to specific stages (e.g., the systematic review).

 Paragraph 6 – I recommend the authors shift this section to the start of the sub-section to firstly indicate the context of COVID-19 in Ireland.

**Methods of trials**

 Please check the correct (past) tense is used throughout. Paragraph 4 states “Assessments are…” such examples recur throughout.

 Page 4, Intervention – what is the evidence-base for the interventional / educational sessions in PIPPRA and MEDRA? This is not clear. Presumably the MEDRA nutritional education sessions were based on previous work.

 Page 5, Amendments to protocol due to COVID-19 pandemic, paragraph 1 – on the final sentence the authors state “The decision-making process for PIPPRA and MEDRA varied”. How exactly? There is an over-reliance on description, without proper explanation or critical appraisal (the latter for the Discussion, not Methods/Results).

 Page 6, paragraph 1, line 2 – ‘virtual platform’. The details of whether and how online telehealth sessions were developed and piloted are missing. Please include. Also, in the following sentences it is mentioned that participants requested to use other online platforms (not just Teams), but equally the reasons are not given. This is important for follow-up and future telehealth care using various online platforms, and in understanding public/patient preferences on digital care.

**Results**

 I recommend that the authors reconsider their use of figures, and instead present PRISMA flow charts for each respective trial. This would limit redundancy (Figure 4 adds very little) and allow the reader to gain oversight in the numbers of participants passing through Recruitment, Assessment, and Intervention. This would also enhance the content, by emphasising the impact that COVID-19 had on rheumatology / clinical research trials.

 Paragraph 3, Assessments – “No assessments occurred…due to participant hesitancy…” what data do you have to support this assertion. Please include on revision or delete this line. Although this is a novel ‘reflective’ study, I feel it is important to support assertions and conclusion with actual data. This can also improve the quality and rigour of the submission.

 Page 7, paragraph 2 – again PRISMA would be much more preferable here. When describing the number of interventions completed, I suggest not using ‘deviations’ or other ambiguous terms, and instead just state whether / how much (%) fewer assessments or intervention sessions were delivered. Does 100% deviation mean that no interventions took place, or all were delivered but online? This is not currently clear.

**Discussion**

 Comparison with existing literature – it would improve the submission with the inclusion of qualitative data, particularly on participant / patient experience (in hesitancy and fear for example). See final sentence of paragraph.

 Page 8, Implications for research and policy, final sentence – “…those who engage with clinical research are likely to be highly interested…” This point requires elaboration, and also consider discussing some of the barriers to clinical-research collaboration if you have observations on these.

**Conclusion**

 Page 9, sentence 2 – 4 – in presenting the conclusion, please also briefly highlight the respective aims of each trial if word count allows. Also, were both trials feasibility studies, as this is mentioned, but in the Introduction the MEDRA appears to have a greater qualitative component and different methodology. Please clarify in revision.

Is the work clearly and accurately presented and does it cite the current literature?

Partly

If applicable, is the statistical analysis and its interpretation appropriate?

I cannot comment. A qualified statistician is required.

Are all the source data underlying the results available to ensure full reproducibility?

No

Is the study design appropriate and is the work technically sound?

Partly

Are the conclusions drawn adequately supported by the results?

Yes

Are sufficient details of methods and analysis provided to allow replication by others?

Partly

Reviewer Expertise:

Musculoskeletal health; physical activity; biomechanics; allied health profession education; occupational therapy; rheumatology

I confirm that I have read this submission and believe that I have an appropriate level of expertise to confirm that it is of an acceptable scientific standard, however I have significant reservations, as outlined above.

# Response

**Published:** 2023-11-06

**Authors:** Louise Louise

**Affiliations:** University of Limerick, Ireland

Abstract

 The use of the word ‘deviations’ is overly vague (especially in the manuscript Results), instead the authors should succinctly state whether the deviation was negative (a decrease) or positive (an increase in this case).

*Thank you for this suggestion. A definition for protocol deviation has been included in the introductory section which hopefully provides clarity on what is meant by deviation. The definition for a protocol deviation is “any departure from the study procedures or treatment plans as specified in the approved protocol.” We have also reviewed the use of the word deviations and revised wording as suggested where appropriate.*

 The Conclusion is weak and reveals little of what was actually found for the individual trials.

*We have considered this feedback and in line with the aim of  the paper the focus of the conclusion was on how the COVID-19 pandemic impacted delivery of the individual trials, as opposed to focusing on what was found for the individual trials.*

 Introduction

 Paragraph 1 – there is an over-reliance on the reference ‘European Commission et al’, which arises three times in the opening paragraph. Also, check that this reference style is correct; should it be‘et al’? I suspect not.

*The number of references has been reduced to two instances and the reference style has been corrected.*

 Paragraph 4 (Protocol design), lines 7-9 – six references are listed to highlight the development work for the PIPPRA trial. Individual references should instead be attributed to specific stages (e.g., the systematic review).

*Individual references have now been attributed to specific stages.*

 Paragraph 6 – I recommend the authors shift this section to the start of the sub-section to firstly indicate the context of COVID-19 in Ireland. *Paragraph 6 has now been moved to earlier in this section as suggested, before Protocol Design.*

 Methods of trials

 Please check the correct (past) tense is used throughout. Paragraph 4 states “Assessments are…” such examples recur throughout.

*Thank you highlighting this. The entire manuscript has been reviewed and updated as suggested. For context at the time of submission to HRB Open the trials were still ongoing and the present tense was used to reflect this.*

 Page 4, Intervention – what is the evidence-base for the interventional / educational sessions in PIPPRA and MEDRA? This is not clear. Presumably the MEDRA nutritional education sessions were based on previous work.

*The evidence base for PIPPRA is as outlined in the Protocol design section. The evidence base for the use of the Mediterranean diet in people who have rheumatoid arthritis is extremely limited, thus the MEDRA study is novel in examining this type of intervention in the RA population.*

 Page 5, Amendments to protocol due to COVID-19 pandemic, paragraph 1 – on the final sentence the authors state “The decision-making process for PIPPRA and MEDRA varied”. How exactly? There is an over-reliance on description, without proper explanation or critical appraisal (the latter for the Discussion, not Methods/Results).

*The decision making process for PIPPRA and MEDRA varied as there were different governance structures within each project and both projects has different requirements in terms of assessments and intervention delivery. We have noted your feedback on description and the need for further explanation and have sought to address this in the Discussion section as highlighted.*

 Page 6, paragraph 1, line 2 – ‘virtual platform’. The details of whether and how online telehealth sessions were developed and piloted are missing. Please include. Also, in the following sentences it is mentioned that participants requested to use other online platforms (not just Teams), but equally the reasons are not given. This is important for follow-up and future telehealth care using various online platforms, and in understanding public/patient preferences on digital care.

*Further details are now provided to clarify this point.*

 Results

 I recommend that the authors reconsider their use of figures, and instead present PRISMA flow charts for each respective trial. This would limit redundancy (Figure 4 adds very little) and allow the reader to gain oversight in the numbers of participants passing through Recruitment, Assessment, and Intervention. This would also enhance the content, by emphasising the impact that COVID-19 had on rheumatology / clinical research trials.

*Thank you for this suggestion. We have included CONSORT flow charts, in line with conducting RCTs, to demonstrate the flow of participants on the recruitment, assessment and intervention phases. We do feel however the Figure 4 is useful in demonstrating the difference in recruitment rates in-person pre-COVID versus online during the COVID-29 pandemic so have opted to retain this figure.*

 Paragraph 3, Assessments – “No assessments occurred…due to participant hesitancy…” what data do you have to support this assertion. Please include on revision or delete this line. Although this is a novel ‘reflective’ study, I feel it is important to support assertions and conclusion with actual data. This can also improve the quality and rigour of the submission.

*Thank you for your query. Participant hesitancy is evidenced by participants declining invitation to attend in-person assessments at that time due to the context of increasing number of COVID-19 cases. Additional information has been included in this section.*

 Page 7, paragraph 2 – again PRISMA would be much more preferable here. When describing the number of interventions completed, I suggest not using ‘deviations’ or other ambiguous terms, and instead just state whether / how much (%) fewer assessments or intervention sessions were delivered. Does 100% deviation mean that no interventions took place, or all were delivered but online? This is not currently clear.

*A definition for protocol deviation has been included in the introductory section which hopefully provides clarity on what is meant by deviation. The definition for a protocol deviation is “any departure from the study procedures or treatment plans as specified in the approved protocol.”*

 Discussion

 Comparison with existing literature – it would improve the submission with the inclusion of qualitative data, particularly on participant / patient experience (in hesitancy and fear for example). See final sentence of paragraph. Reference to how fear of COVID-19 impact access to healthcare is now included.

 Page 8, Implications for research and policy, final sentence – “…those who engage with clinical research are likely to be highly interested…” This point requires elaboration, and also consider discussing some of the barriers to clinical-research collaboration if you have observations on these.

*An additional reference has been included to provide evidence towards the former point. An additional statement, with evidence from the rheumatology population is now included to address the latter point.*

 Conclusion

 Page 9, sentence 2 – 4 – in presenting the conclusion, please also briefly highlight the respective aims of each trial if word count allows. Also, were both trials feasibility studies, as this is mentioned, but in the Introduction the MEDRA appears to have a greater qualitative component and different methodology. Please clarify in revision.

*The aims of both studies have now been included. Both studies had slight differences in methodologies which were introduced due to the COVID-19 pandemic.*

# Reviewer report: Reviewer response for version 1

**DOI:** 10.21956/hrbopenres.14458.r29514

**Published:** 2021-06-08

**Authors:** Hannah Durand

**Affiliations:** 1 School of Psychology, National University of Ireland, Galway, Ireland

Thank you for the opportunity to review this manuscript, entitled "The impact of COVID-19 on clinical research: the PIPPRA and MEDRA experience." This article describes how the COVID-19 pandemic and resultant public health restrictions and guidance for the conduct of clinical trials impacted two clinical rheumatology research trials - the Physiotherapist-led Intervention to Promote Physical Activity in Rheumatoid Arthritis (PIPPRA) and the MEDiterranean diet in Rheumatoid Arthritis (MEDRA) projects. Publication of such reports is of vital importance. Such work contributes enormously to the clinical trials literature and enhances transparency around research conducted during COVID-19, which has in many cases necessitated deviations from *a priori* research protocols and procedures. Published experiences of trialists operating during the COVID-19 pandemic may help future researchers, funders and clinical teams to adapt to this and future pandemics in a way that limits research waste. The current focus on two trials for rheumatology patients is important, given the nature of the disease and the elevated risk of serious illness and death from COVID-19 infection due to immunosuppression among this group . 

 The authors present a clearly articulated and structured manuscript. The following comments are intended only to improve the quality and clarity of this submission. 

**Introduction**

 The third paragraph could be reorganised for clarity. I found myself scrolling up to see had I missed the first mention that this article is about the impact of COVID-19 and its restrictions on two trials. An additional sentence at the start of the paragraph setting up the context of the article would be helpful. (e.g., final line of the Intro: "This paper seeks to outline the experience of two clinical rheumatology research projects in the Republic of Ireland across the first 10 months (March 2020 – December 2020) of the COVID-19 global pandemic.") 

**Protocol Design**

 It is not clear what is meant by "test the feasibility of the intervention on secondary disease focused outcome measures." From this phrasing it seems like what is being tested here is preliminary efficacy?

**Methods of Trials**

 For PIPPRA, participants must have a rheumatologist confirmed diagnosis of RA. For MEDRA, participants must have a definite diagnosis of RA according to American College of Rheumatology (ACR)/European League Against Rheumatism (EULAR) criteria. I am interested to know if these criteria are meaningfully different (e.g., are rheumatologists in Ireland likely to use another set of criteria in making a diagnosis of RA?). Though these are independent trials it could be useful to know if there are potentially any clinical differences between the two trial participant groups. If so, please briefly comment. If not, please ignore.  

**Intervention**

 Should "project research assistant (AM, TP)." be pluralised? 

**PIPPRA Amendments**

 Was the cost of participants' posting the ActivPAL to the assessor covered and, if so, how (free post label, reimbursement?).  

**Discussion**

 I am struck that one trial is part of an investigator led project and the other forms part of a PhD project. I would expect that the implications of COVID-19 on clinical trials for PIs versus PhD students / candidates  (and indeed other early career researchers) are quite different, particularly where the adaptations turn out to  be ineffective. Could the authors comment on whether they perceived any unique challenges to adapting trial protocols to the pandemic restrictions/participant preferences for the HRB funded project versus the PhD project?

Is the work clearly and accurately presented and does it cite the current literature?

Yes

If applicable, is the statistical analysis and its interpretation appropriate?

Not applicable

Are all the source data underlying the results available to ensure full reproducibility?

Yes

Is the study design appropriate and is the work technically sound?

Yes

Are the conclusions drawn adequately supported by the results?

Yes

Are sufficient details of methods and analysis provided to allow replication by others?

Yes

Reviewer Expertise:

health psychology, behaviour change, clinical health research

I confirm that I have read this submission and believe that I have an appropriate level of expertise to confirm that it is of an acceptable scientific standard.

# Response

**Published:** 2023-11-06

**Authors:** Louise Louise

**Affiliations:** University of Limerick, Ireland

**Introduction**

 The third paragraph could be reorganised for clarity. I found myself scrolling up to see had I missed the first mention that this article is about the impact of COVID-19 and its restrictions on two trials. An additional sentence at the start of the paragraph setting up the context of the article would be helpful. (e.g., final line of the Intro: "This paper seeks to outline the experience of two clinical rheumatology research projects in the Republic of Ireland across the first 10 months (March 2020 – December 2020) of the COVID-19 global pandemic.")

*To improve clarity the paragraph on the impact of COVID-19 on the clinical research community has been moved to the third paragraph so that the concept is introduced earlier in the paper. Thank you for the suggested sentence for inclusion, which is now included in the paper.*

**Protocol Design**

 It is not clear what is meant by "test the feasibility of the intervention on secondary disease focused outcome measures." From this phrasing it seems like what is being tested here is preliminary efficacy? *This sentence has been revised to provide clarity.*

**Methods of Trials**

 For PIPPRA, participants must have a rheumatologist confirmed diagnosis of RA. For MEDRA, participants must have a definite diagnosis of RA according to American College of Rheumatology (ACR)/European League Against Rheumatism (EULAR) criteria. I am interested to know if these criteria are meaningfully different (e.g., are rheumatologists in Ireland likely to use another set of criteria in making a diagnosis of RA?). Though these are independent trials it could be useful to know if there are potentially any clinical differences between the two trial participant groups. If so, please briefly comment. If not, please ignore.

*Thank you for your query.  Rheumatologists in Ireland use the American College of Rheumatology (ACR)/European League Against Rheumatism (EULAR) criteria for diagnosis.*

**Intervention**

 Should "project research assistant (AM, TP)." be pluralised?

*Yes, thank you highlighting this error. This has now been edited.*

**PIPPRA Amendments**

 Was the cost of participants' posting the ActivPAL to the assessor covered and, if so, how (free post label, reimbursement?).

*Participants were reimbursed for postal charges via vouchers. This information has now been included.*

**Discussion**

 I am struck that one trial is part of an investigator led project and the other forms part of a PhD project. I would expect that the implications of COVID-19 on clinical trials for PIs versus PhD students / candidates (and indeed other early career researchers) are quite different, particularly where the adaptations turn out to be ineffective. Could the authors comment on whether they perceived any unique challenges to adapting trial protocols to the pandemic restrictions/participant preferences for the HRB funded project versus the PhD project?

*In the case of PIPPRA and MEDRA projects there were no unique challenges in the HRB funded vs PhD project. This can be attributed to the fact that both projects had small operational teams (PIPPRA – PI, Postdoc, RA; MEDRA – PhD student, Lead Supervisor). Of note is that the PIPPRA PI and Postdoc were also MEDRA PhD supervisors which provided good problem solving and learning opportunities in adapting to the challenges provided by the COVID-19 pandemic. Both the PIPPRA and MEDRA funding was supported by the relevant funders who provided opportunities to extend project timelines to support completion of both projects.*
